# 16S rRNA Gene Survey of Microbial Communities in Winogradsky Columns

**DOI:** 10.1371/journal.pone.0104134

**Published:** 2014-08-07

**Authors:** Ethan A. Rundell, Lois M. Banta, Doyle V. Ward, Corey D. Watts, Bruce Birren, David J. Esteban

**Affiliations:** 1 Department of Biology, Vassar College, Poughkeepsie, New York, United States of America; 2 Department of Biology, Williams College, Williamstown, Massachusetts, United States of America; 3 Genome Sequencing Center, Broad Institute, Cambridge, Massachusetts, United States of America; Argonne National Laboratory, United States of America

## Abstract

A Winogradsky column is a clear glass or plastic column filled with enriched sediment. Over time, microbial communities in the sediment grow in a stratified ecosystem with an oxic top layer and anoxic sub-surface layers. Winogradsky columns have been used extensively to demonstrate microbial nutrient cycling and metabolic diversity in undergraduate microbiology labs. In this study, we used high-throughput 16s rRNA gene sequencing to investigate the microbial diversity of Winogradsky columns. Specifically, we tested the impact of sediment source, supplemental cellulose source, and depth within the column, on microbial community structure. We found that the Winogradsky columns were highly diverse communities but are dominated by three phyla: Proteobacteria, Bacteroidetes, and Firmicutes. The community is structured by a founding population dependent on the source of sediment used to prepare the columns and is differentiated by depth within the column. Numerous biomarkers were identified distinguishing sample depth, including Cyanobacteria, Alphaproteobacteria, and Betaproteobacteria as biomarkers of the soil-water interface, and Clostridia as a biomarker of the deepest depth. Supplemental cellulose source impacted community structure but less strongly than depth and sediment source. In columns dominated by Firmicutes, the family Peptococcaceae was the most abundant sulfate reducer, while in columns abundant in Proteobacteria, several Deltaproteobacteria families, including Desulfobacteraceae, were found, showing that different taxonomic groups carry out sulfur cycling in different columns. This study brings this historical method for enrichment culture of chemolithotrophs and other soil bacteria into the modern era of microbiology and demonstrates the potential of the Winogradsky column as a model system for investigating the effect of environmental variables on soil microbial communities.

## Introduction

Sergei Winogradsky is a founder of modern microbiology and microbial ecology, and is credited with the discovery of chemolithotrophy [1], [2]. His name is familiar to microbiologists and microbiology students as the originator of Winogradsky columns, commonly used in microbiology education to demonstrate microbial diversity and nutrient cycling. Traditionally, these enrichment cultures are created by culturing mud or sediment in a transparent column with a cellulose source and a source of sulfate and/or other nutrients. Over time, chemical gradients result in a vertical distribution of unique niches for microbial growth. In addition to an oxic niche at the top and anoxic sub-surface niches, the production of H_2_S generated by sulfur reducing microbes results in high H_2_S in anoxic layers, which diffuses up towards the oxic layer. This stratification in the column leads to the growth of different microbes at different depths, exemplified by the growth of pigmented microorganisms, including phototrophs, producing visible layers. Requiring only the input of light, the Winogradsky column establishes a structured microbial ecosystem, carrying out essential nutrient cycles including the carbon, nitrogen and sulfur cycles.

In nature, sediment and soil microbial communities contribute critically to biogeochemical cycling and the degradation of pollutants and toxins. The structure and function of these communities may be impacted by differences in oxygen concentration, water levels, nutrient levels, pH, and other factors [3]–[7] that make difficult the study of such communities and their responses to experimental manipulation. By contrast, Winogradsky columns are easy to create, replicate, and control. Thus they can be powerful models for discovering the impact of specific variables on stratified microbial communities as well as studying nutrient cycling and bioremediation.

Past studies have used Winogradsky columns in the study of soluble-reactive phosphate generation, bioremediation, and biohydrogen production by microbes [8]–[10]. Winogradsky columns (and similarly designed Winogradsky plates) have also been used as enrichment cultures for microbial groups including phototrophs and sulfur cycle microbes [11]–[13]. Novel microorganisms have been isolated and classified from columns [14]–[16]. Similarly, microcosms of rice paddy soils have been used to describe microbial communities along oxygen gradients [3], however these differ from Winogradsky columns in that they are incubated in the dark.

Soil microbial communities are exceptionally diverse and contain numerous species refractory to growth under laboratory culture conditions. Therefore, culture-based methods inaccurately represent natural microbial community composition [17]. Sequence-based surveys of environmental samples, using high throughput techniques, can reveal a more complete picture of microbial diversity than is possible through culture-based methods. Despite the fact that Winogradsky columns are widely used to demonstrate microbial diversity and have the potential for application to studies of microbial community dynamics in stratified ecosystems, to our knowledge they have never been evaluated using high-throughput sequencing methods.

Here we present the application of high-throughput sequencing to Winogradsky column microbial populations. We conducted a 16S rRNA gene survey of Winogradsky columns in order to investigate the diversity and structure of the communities present in these enrichment cultures, and the influence of environmental variables on these populations. We investigated the effects of depth, sediment source, and supplemental organic carbon source on microbial community structure. We demonstrate that Winogradsky column microbial communities are exceptionally diverse and that the community structure is determined by a founder effect from the sediment source used to create the column, which is further stratified by depth within the column.

## Methods and Materials

### Column Preparation

Twelve Winogradsky columns were created from two small ponds located near the campus of Williams College in Williamstown, Massachusetts: Buxton Pond (N 42.70° (42° 42′ 15′′) W 73.2114° (73° 12′ 41′′) and Eph's Pond (N 42.7201° (42° 43′ 12′′) W 73.1975° (73° 11′ 51′′). Buxton Pond is an artificial pond fed by ground water, and has an overflow that is piped to the east under Gale Road to Christmas Brook. It is in close proximity to a road and is entirely shaded. Eph's Pond is farther from roads and receives more direct sunlight than Buxton Pond. Prior to 1912, the present location of Eph's Pond was marsh and swampy land lacking in standing water. The pond was formed upon construction of a road that impeded the drainage from the center of the Williams College campus toward the Hoosic River. The hydrology of the pond has inputs from both nearby springs as well as storm sewers to the south, while the output is directed through a 24-inch pipe at the pond's northwest corner. Currently Eph's Pond has extensive cattail, sedge, and rush marsh bordering the declining extent of open water in a shallow pond (<1 meter depth) that is rapidly filling in with sediment. Both sampled sites are located on private land. The samples taken for this study qualify for the "minor activities" exemption to the Massachusetts Wetlands Protection Act (310 Code of Massachusetts Regulations 10.00, section 10.58(6)).

Sediment was collected from near the edge of each pond (under approximately 15–30 cm of water) in late October 2008; sticks and other large debris were removed. The pond sludge was mixed to homogeneity with 2% by weight MgSO_4_, 2% by weight CaCO_3_, and an equivalent volume of either oak and maple leaf litter or chopped vegetables (organically grown lettuce and red peppers from local farms) as supplemental organic carbon sources. Each mixture together with a small amount of pond water was packed into Plexiglas columns (5.5 cm diameter, 18 cm height, Carolina Biologicals), taking care to force out any trapped air. The packed sediment was overlaid with 2–3 cm of pond water, and the columns were covered tightly with saran wrap, which was secured with a rubber band. Three replicate columns were created for each condition (Eph's Pond with vegetable scraps, Eph's Pond with leaf litter, Buxton Pond with vegetable scraps and Buxton Pond with leaf litter). Columns were incubated under mixed incandescent and fluorescent light (130 umoles/m2/sec) in a Conviron growth chamber at room temperature for 18 weeks. Samples were collected from each column at the soil water interface (SWI) and at 4, 8, and 12 cm below it. To collect samples, holes were drilled in to the sides of each column with a 0.25 inch drill bit, which was carefully washed between uses, and sediment was scooped with a small scoopula or drawn with a syringe. Additional samples from some columns were taken at other depths. Some samples were taken from the top surface by scraping the biofilm at the soil-water interface with a loop; these are referred to as “top surface” samples to distinguish them from SWI samples collected by drilling. Samples were pelleted by centrifugation at 13,000 rcf for 30 s. At least 0.1 g of soil was obtained after liquid removal, and frozen at −20°C until used. Samples from column replicates 1 and 2 were extracted at Williams College, and samples from column replicate 3 were extracted at Vassar College.

### DNA Extraction, Amplification and Sequencing

DNA extraction was performed using a MoBio Powersoil DNA isolation kit (MoBio, CA) following the manufacturer's directions. The V4 region of the 16S rRNA gene was amplified using PCR following the protocol described in [18] and 515F and 806R primers targeting the V4 region [19]. Sequencing was performed using Illumina's MiSeq to generate paired-end reads. Sequences were deposited in MG-RAST (ID: mgp7374) and NCBI (BioProject ID: PRJNA234104).

### Quality filtering and OTU picking

SpliceReads, a part of ALLPATHS software [20] was used for assembly of paired-end reads. Filtering, chimera-checking, OTU picking, and taxonomy assignment were conducted using Quantitiative Insights in Microbial Ecology (QIIME, v1.6.0) [21]. Briefly, *de novo* OTU clustering at 97% identity was performed using usearch (default settings) [22] and with greengenes OTUs for chimera detection. A representative sequence for each OTU was selected and an OTU table was generated. Taxonomic identities were assigned using the RDP Classifier [23] retrained with greengenes taxonomy in QIIME using default settings. Additional filtering for sequence errors was performed by removing OTUs appearing in fewer than 2 samples or containing fewer than 50 sequences across all samples using QIIME's filter_otus_from_otu_table.py script.

### Diversity analysis

Alpha diversity was calculated using the Shannon index, OTU richness, and Berger-Parker Dominance index in QIIME. Rarefaction curves were generated by repeated (10 times) subsampling of 10 to 800 sequences, with steps of 79 sequences. We considered only those samples with four or more replicates; that is, samples scraped from the top surface, the SWI, and 4, 8, and 12 cm below the SWI.

To test for significant differences in alpha diversity, QIIME's compare_alpha_diversity.py script was used to run nonparametric two-sample t-tests. The default number of monte-carlo permutations (999) were used to calculate p-values in the nonparametric t-tests, and a significance threshold of p<0.05 was used.

Phylogenetic analysis was performed by aligning representative sequences with PyNAST, followed by alignment filtering to remove non-informative positions, using default settings in QIIME. Phylogenetic beta diversity was calculated using both unweighted and weighted UNIFRAC [24], [25]. To estimate the support for beta diversity results, rarefaction was used to generate 100 800-sequence subsamples from each sample. Principal coordinate plots and bootstrapped consensus UPGMA trees were generated from these rarefied distance matrices. Chi-square, Canberra, Pearson, and Spearman indices were also calculated in QIIME. To evaluate statistical significance of beta diversity results, we used nonparametric two-sample t-tests on weighted UNIFRAC distances calculated from the entire dataset (without rarefaction), with the default number of Monte Carlo permutations (999). A p value of less than 0.05 was considered a cutoff point for significant difference. All samples, including those with few or no replicates (that is, samples taken at 3 cm and 6 cm) were included in beta diversity analyses.

### Comparison to other microbial communities

To compare Winogradsky column microbial communities to previously sequenced uncultured communities, we used MG-RAST [26]. In order to compare entire Winogradsky columns to uncultured communities we pooled data from the same columns. All steps, including quality filtering, assembly and normalization, used default settings. To minimize biases caused by the use of different sequencing techniques, we restricted our analysis to a comparison to paired-end sequences data obtained by Caporaso et al [18], which used identical primers but had shorter reads. We used the taxonomy-based Bray-Curtis beta diversity index in MG-RAST to generate principal coordinate plots.

### Biomarker analysis

Linear discriminant effect size (LEfSe) [27] was used to identify microbial biomarkers for depth, sediment source, or supplemental organic carbon source. We included only those samples from the SWI, 4, 8, and 12 cm depths due to the lower number of replicates from other depths. Unclassified terminal features were removed from the data, leaving them in at higher, classified ranks. For example, OTUs unclassified at the genus level were removed from the genus rank, but were kept in the family and higher ranks. In this way, unclassified OTUs contribute to biomarker analysis at higher taxonomic ranks (where they are classified) but unclassified organisms (difficult to resolve and treat as taxonomic groups based on sequence information alone) are not classified as biomarkers. All settings were set to default with two exceptions. Depth biomarkers were identified using the less strict (one-against-all) setting, and sediment source biomarkers were identified with the requirement that only samples of the same depth be compared (thus to be considered a sediment source biomarker a microbe had to be consistently higher in abundance in one sediment source in each corresponding layer).

## Results

### Description of Columns and Samples

**Figure 1:**
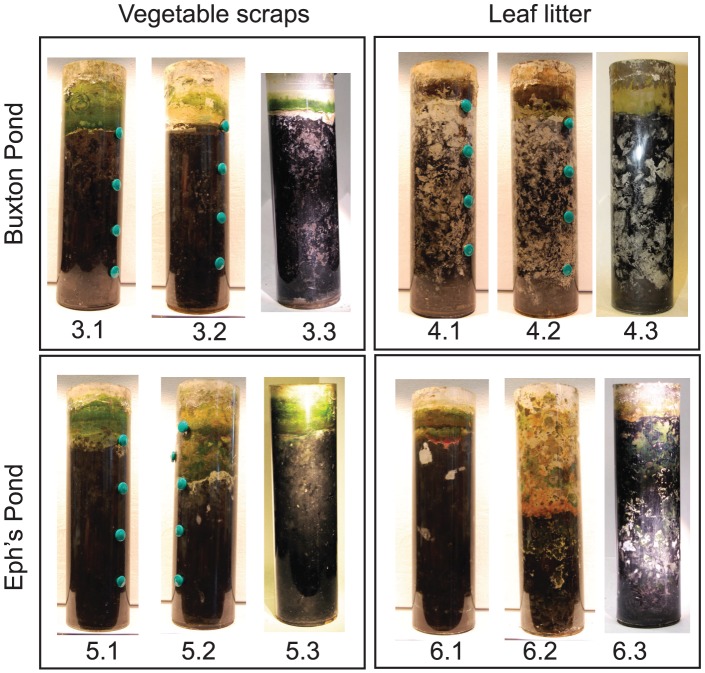
Winogradsky columns. Triplicate columns of each condition were prepared. Photographs were taken following sample collection. The teal plugs covering the holes drilled can be seen in some columns; the topmost plug is at the SWI. Column numbers are shown below the image.

Winogradsky columns often have several bands of colors at different depths. All columns displayed bands of color at the soil water interface (SWI). At subsurface levels, most did not show the banding patterns commonly seen in Winogradsky columns, although patches of numerous different colors were seen throughout (Figure 1). Replicate columns did not necessarily look the same, but typically looked more like each other than columns with different sediment or supplemental organic carbon sources. Samples were taken by scraping the tops of some columns, and drilling holes at the SWI and at 4 cm intervals below in all columns. The local color of the sampling site was recorded (Table S1). The samples were quite loose, and the sediment was grey except for the 12 cm samples, which were black. Plating of samples on each of several standard bacteriological media yielded cultivable microbial populations that were strikingly similar across all fractions from a given column as well as across columns, with only a small number of distinct morphologies visible on each type of media (data not shown).

### Sequence Data

Paired end reads of 175 bp were generated by Illumina sequencing. After assembly of paired end reads, there were 642,384 assembled sequences in 54 samples with an average read length of 253 bp. High throughput sequencing datasets are likely to contain sequencing errors and chimeric PCR products, so we applied chimera filtering in USEARCH and removed extremely rare sequences in QIIME. 135,155 sequences were removed in this process, leaving 507,229. The mean number of sequences per sample was 9393.

### Alpha Diversity

In total, 31 phyla were present across all samples with an average of 23 phyla per sample (range: 12 to 30), indicating exceptional diversity (Figure S1). However, the column communities were dominated heavily by members of three abundant phyla (Proteobacteria, Bacteroidetes and Firmicutes) representing an average of 75% of each sample. 414 genera were identified across all columns with an average of 245 genera per sample (range: 102 to 323). The majority of these microbial genera (99%) appeared in three or more samples and 90% appeared in 10 or more.

A critical component of evaluating microbial community structure is the consideration of alpha diversity, or within-community diversity. The Shannon index measures the diversity of communities, taking into account their richness (or the number of distinct taxa that are present) and the evenness of taxon distribution. To investigate whether sequencing depth captured the diversity of our samples, and to assess the impact of soil depth, sediment source, and organic carbon source on Winogradsky column diversity, we generated rarefaction curves for the Shannon index.

**Figure 2:**
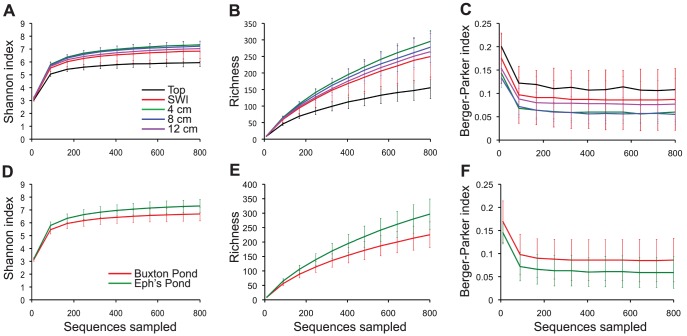
Alpha diversity of Winogradsky column samples. Alpha diversity indices were calculated on rarefied samples. Samples were pooled by layer (A,B,C) or by sediment source (D,E,F) and the average Shannon index (A,D), richness (B,F) and Berger-Parker dominance index (C,F) were calculated. Error bars represent standard error for each category. Significant differences were seen between the Shannon index of top surface samples and samples taken from 4, 8, and 12 cm below the surface (A, non-parametric t-test, p = 0.021) and between sediment sources (D, nonparametric t-test, p = 0.001). Top surface samples were significantly less rich than samples at 4, 8, and 12 cm below the SWI (B, non-parametric t-test, p = 0.021 for surface vs. 4 cm, p cm, p = 0.021 surface vs. 8 cm, and p cm, and p = 0.042 for surface vs. 12 cm cm) and Eph's Pond columns are significantly more rich than Buxton Pond columns (E, non-parametric t-test, p = 0.002). Buxton Pond columns were significantly more dominated by single taxa than Eph's Pond columns (F, non-parametric t-test, p = 0.015).

The Shannon index rarefaction curves approached asymptotes, indicating that sampling depth was sufficient to capture the overall diversity of Winogradsky column microbial communities (Figure 2 A and D). Winogradsky column microbial diversity is exceptionally high (the average Shannon index value at a sampling depth of 800 sequences was 6.19) and both depth and sediment source impacted the diversity of Winogradsky column communities. Top surface samples were the least diverse (Figure 2 A). Significant differences were seen between top surface samples and samples taken from 4, 8, and 12 cm below the surface (non-parametric t-test, p = 0.021). Communities in Eph's Pond columns were significantly more diverse than those in Buxton Pond columns (Figure 2 D, nonparametric t-test, p = 0.001), but organic carbon source had no effect on diversity (p = 0.633, data not shown).

Richness is a count of the number of taxa present in a community. Rarefaction curves of OTU richness did not approach asymptotes (Figure 2 B and E), even when extended to maximum sampling depth (data not shown). Rarefaction of singles (OTUs appearing only once in a sample) also continued to rise with increased sampling depth (data not shown). Taken together, this suggests that either sequencing depth was insufficient to capture all rare organisms or that sequencing errors were contributing to apparent diversity. Top surface samples were significantly less rich than samples at 4, 8, and 12 cm below the SWI (p = 0.021 for surface vs. 4 cm, p = 0.021 surface vs. 8 cm, and p = 0.042 for surface vs. 12 cm) (Figure 2B). Samples taken from Eph's Pond columns were significantly more rich than those from Buxton Pond columns (Figure 2E) (p = 0.002). Organic carbon source did not have an effect on richness (p = 0.742, data not shown).

The Winogradsky column microbial communities had few abundant genera and numerous rare genera. Only 5–8% of genera were present in greater than 1% abundance, 23–34% of genera were present in 0.1–0.99% abundance, and the remaining 61–78% were very rare, present at less than 0.1% abundance (Table 1). This shows a fairly uneven distribution of taxa, so we applied the Berger-Parker dominance index, a measure of the relative abundance of the most abundant member of a microbial community. Dominance did not significantly differ by depth (Figure 2C), but Buxton Pond columns were significantly more dominated by single taxa than Eph's Pond columns (p = 0.015, Figure 2F). Organic carbon source did not have an effect on dominance (p = 0.467, data not shown).

**Table 1:** Distribution of abundant and rare genera.

	Sample						
Abundance range	All	Eph's	Buxton	SWI	4 cm	8 cm	12 cm
>10%	0 (0)	1 (0)	0 (0)	0 (0)	1 (0)	0 (0)	0 (0)
1–10%	24 (6)	23 (6)	30 (8)	25 (6)	19 (5)	24 (6)	25 (7)
0.1–0.99	135 (33)	135 (33)	94 (24)	126 (31)	134 (34)	104 (26)	87 (23)
<0.1%	255 (62)	246 (61)	273 (69)	257 (63)	239 (61)	266 (68)	296 (78)
Total	414 (100)	405 (100)	397 (100)	408 (100)	393 (100)	394 (100)	381 (100)

Samples were categorized by depth or sediment source and the average abundance of each genus was calculated. Number of genera (percent) is shown.

### Beta Diversity

To investigate the impact of column depth, sediment source and organic carbon source on beta diversity (between-sample diversity), we evaluated our samples using UNIFRAC, a metric evaluating the phylogenetic distance between pairs of communities based on the fraction of total phylogenetic tree branch length unique to both communities [25]. We used both unweighted UNIFRAC, which uses only OTU presence/absence data, and weighted UNIFRAC, which incorporates relative abundance information in to its calculation of phylogenetic distance [24].

**Figure 3:**
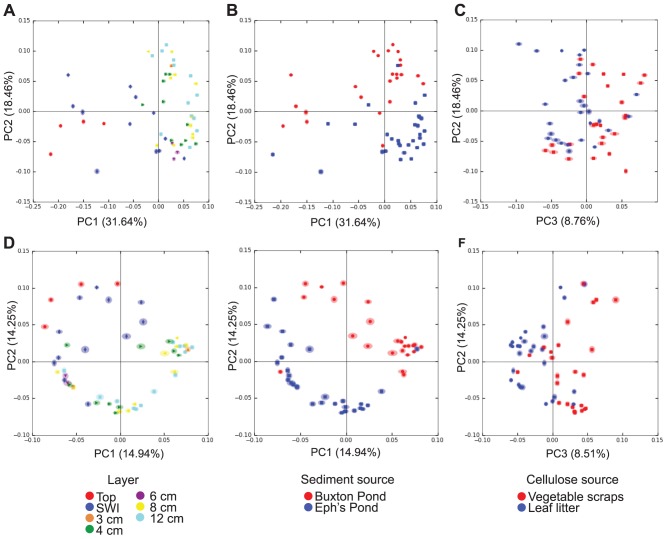
Principal coordinate analysis of Winogradsky column beta diversity. Principal Coordinate plots of weighted UNIFRAC (A,B,C) and unweighted UNIFRAC (D,E,F) results were generated and colored by depth (A,D), sediment source (B,E) or cellulose source (C,F). 100 rarefactions were conducted at a depth of 800 sequences per sample to estimate robustness of beta diversity patterns. Shading around each point represents interquartile range of that point's placement as calculated based on rarefied PCoA.

Both depth and sediment source substantially impacted Winogradsky column microbial communities, while organic carbon source had a less pronounced effect (Figure 3). Samples scraped off the surface of columns and taken at the SWI were clustered separately from those taken at lower depths along a principal coordinate axis (PCoA) explaining 31.64% of the variation in the data when weighted UNIFRAC was considered (Figure 3A). Unweighted UNIFRAC also indicated separation based on depth; however it occurred along a principal coordinate axis explaining less of the variation in the data (14.94%) (Figure 3D). The compositions of the subsurface layers (4 cm, 8 cm, and 12 cm) were not distinguishable from one another in PCoA plots.

Samples clustered by sediment source in PCoA plots generated from both weighted and unweighted UNIFRAC, indicating a strong influence of sediment source on the column communities (Figure 3B and 3E). In weighted UNIFRAC analysis, samples clustered by sediment source along a second principal coordinate explaining 18.46% of the variation in the data. A third principal coordinate axis in both weighted and unweighted UNIFRAC less clearly separated samples by organic carbon source (Figure 3C and 3F). This axis explained less of the variation in the data (about 9% in weighted and unweighted UNIFRAC).

**Figure 4:**
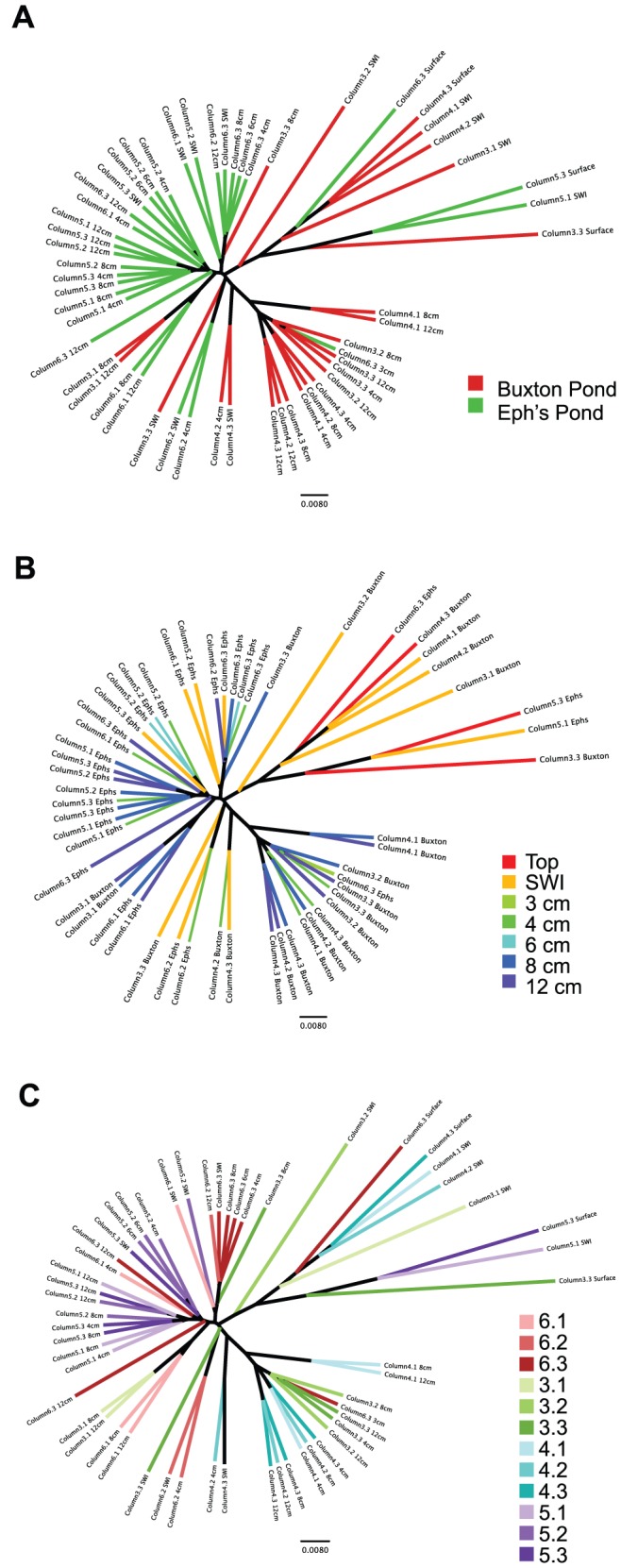
UPGMA trees of all Winogradsky column samples show separation by depth and sediment source. Rarefied weighted UNIFRAC results were used to generate a consensus UPGMA tree. Samples are colored by (A) sediment source, (B) depth, and (C) column.

The substantial impact of both depth and sediment source on community structure in columns was further verified by nonparametric two-sample t-tests on within group vs. between group weighted UNIFRAC distances. These tests indicated that both depth and sediment source significantly impacted beta diversity (within vs. between depth nonparametric p value  = 0.001 and within sediment source vs. between sediment source nonparametric p-value  = 0.001). Organic carbon source also significantly impacted beta diversity, but less so (nonparametric p-value  = 0.01), which is consistent with the less pronounced separation of the samples by organic carbon source in PCoA. The effect of both depth and sediment source on Winogradsky column beta diversity was also investigated by generating a consensus UPGMA tree from rarefied weighted UNIFRAC results. Two major clades were present, each comprised mainly of samples from a single sediment source (Figure 4A). Depth-based clades were not apparent; however, surface and SWI samples tended to branch separately from deeper level samples (Figure 4B).

Throughout the tree, we noted that different layers from the same column tend to be closely related, demonstrating similarity of the community within a whole column (Figure 4C). Replicates were seen to cluster together and we noted that column samples that were visibly similar had similar communities (Table S1). The 4 cm, 8 cm, and 12 cm samples of replicate columns 4.1, 4.2, and 4.3 (Buxton Pond, leaf litter) form a large clade along with several other Buxton Pond column samples, all of which were primarily black with flecks or patches of grey and white. The SWI, 4 cm, 6 cm, and 8 cm samples of column 6.3 (Eph's Pond, leaf litter) cluster together, and all are described as having various shades of green. The sample descriptions and overall appearance of columns 6.1 and 6.2 are quite different from that of 6.3, and this is also reflected in more divergent placement of these samples on the tree. Several triplicates with similar or identical descriptions cluster together. The 12 cm samples from columns 5.1, 5.2 and 5.3 (Eph's Pond, vegetable scraps) form a small clade, and the 8 cm samples form a clade along with two other column 5 replicate samples. These are all described as being black with grey flecks or patches. In contrast, the 4 cm samples from same columns do not cluster closely and have differing descriptions. The 4 cm samples from columns 6.1, 6.2, and 6.3 also do not cluster together and have different sample descriptions.

There are numerous analysis methods available to compare microbial communities. Certain indices are well suited to detecting community differences in samples distributed along an environmental gradient, while others are more appropriate for identifying factors that contribute to sample clustering. We selected four taxonomy-based beta diversity metrics [28] based on their appropriateness for revealing microbial community differences along an environmental gradient, such as an oxygen gradient (Chi-Square and Pearson), or clusters, such as would be expected from sediment source effects (Spearman and Canberra). All analyses showed sample clustering by sediment source, while by depth, subsurface samples clustered separately from SWI and top surface samples (data not shown) supporting the results obtained with UNIFRAC analysis.

Finally, statistical analyses of weighted UNIFRAC distances by two-sample t-testing revealed that replicate samples tended to be more similar to one another than non-replicates (p = 0.001). Replicate samples tended to cluster together in principal coordinate analyses and UPGMA trees, though some heterogeneity was apparent, particularly in samples taken at the SWI (data not shown). The heterogeneity of SWI samples was confirmed by nonparametric two-sample t-tests, which indicated that replicate samples from the SWI were less similar to one another than other same-depth pairs of samples (p = 0.001). We also found no differences between samples extracted and prepared at Vassar College vs Williams College (data not shown).

### Microbial Biomarkers

**Figure 5:**
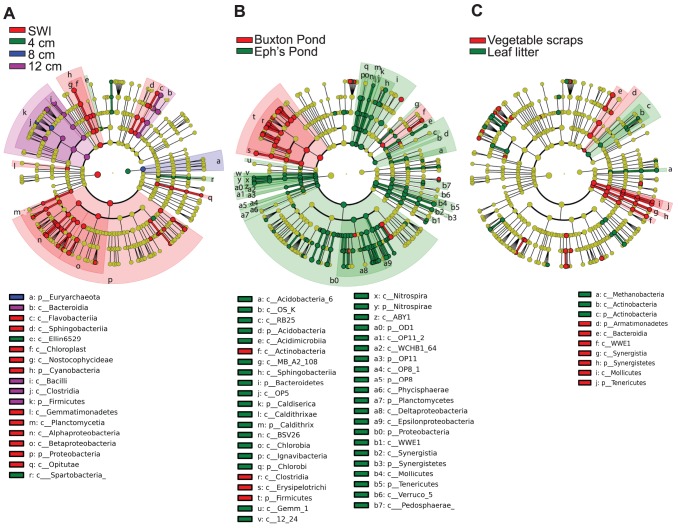
Cladograms of biomarkers for depth, sediment source, and cellulose source. LEfSe was used to identify biomarkers that discriminate (A) depth, (B) sediment source, and (C) cellulose source in Winogradsky columns. Concentric rings from outside in are genus, family, order, class and phylum, with the two central circles for bacteria and archaea. All taxa present are shown, with colored circles representing biomarkers and yellow circles representing non-discriminating taxa. Shaded areas show all taxa below phylum or class biomarkers. For clarity, only biomarkers at the phylum and class levels are labeled.

Given the clear effects of both sediment source and depth on communities of Winogradsky columns, we next sought to determine the taxonomic groups that consistently and significantly differed in abundance in accordance with these variables. We used LEfSe, to identify biomarkers consistently varying in abundance according to depth or sediment source [27]. Our findings indicate that these Winogradsky columns are characterized by major phylum and class-level differences in microbial abundance associated with both depth and sediment source, and (to a lesser extent) organic carbon source (Figure 5). Across all taxonomic levels, 87 biomarkers were associated with depth, 194 were associated with sediment source, and 73 were associated with organic carbon source (Table S1).

**Figure 6:**
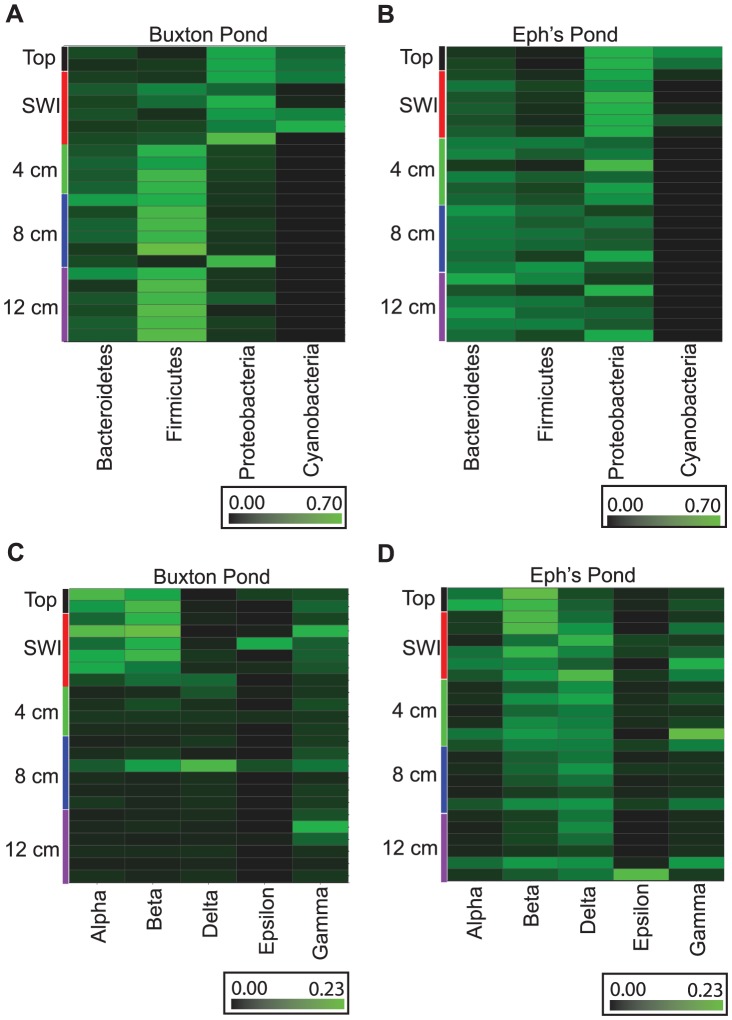
Heatmaps of abundant taxa in Winogradsky columns. Relative abundance of the six most abundant phyla is shown for (A) Buxton Pond and (B) Eph's Pond, and of Proteobacteria classes in (C) Buxton Pond and (D) Eph's Pond. Samples are organized by column depth from top to bottom.

The Proteobacteria, a biomarker for SWI, were highest in abundance at the tops of columns and decrease in abundance with increasing depth (Figure 6A and 6B). Interestingly, the degree to which Proteobacteria abundance decreased with depth differed substantially in a sediment-source dependent manner. In Buxton Pond columns Proteobacteria abundance dropped sharply below the SWI while in Eph's Pond columns abundance remained higher at greater depths. Based on this pattern we investigated the distribution patterns of the abundant classes in this diverse phylum (Figure 6C and 6D). Alphaproteobacteria and Betaproteobacteria, both biomarkers for SWI (Figure 5A), showed the same pattern in Buxton Pond columns as the phylum level Proteobacteria pattern, with a sharp decrease below the SWI (Figure 6C). In Eph's Pond columns, the Alphaproteobacteria were less abundant and concentrated in the upper layers, but the Betaproteobacteria and Deltaproteobacteria, while most abundant in the upper layers, were also abundant at greater depths (Figure 6D). Deltaproteobacteria were a biomarker for Eph's Pond (Figure 5B).

Firmicutes and class Bacteroidia were biomarkers for the lowest (12 cm) depth (Figure 5A). Both increased in abundance with increasing depth (Figure 6A and 6B). Below the SWI, Buxton Pond columns were dominated by Firmicutes, a biomarker for Buxton Pond, while Eph's Pond columns had abundant Proteobacteria, Firmicutes and Bacteroidetes. The Firmicutes population in both columns was largely dominated by the class Clostridia and order Clostridiales, while the Bacteroidetes were dominated by class Bacteroidia and order Bacteroidales (data not shown).

Other biomarkers included the Cyanobacteria, which only appeared in SWI samples, and the order Methylococcales and the Archaeal phylum Euryarchaeota, which were biomarkers for samples taken at 4 cm and 8 cm respectively (Figure 5A and Table S2). Interestingly, microbes often found in gut microbiota were identified as biomarkers for Buxton Pond – these were the genus *Bacteroides*, the order Enterobacteriales, and the genus *Ruminococcus* (Table S2). Additionally, several microbes associated with dehalogenation, degradation of aromatic compounds, and the degradation of industrial toxins were also identified including the genus *Dehalobacter* (higher in abundance in Buxton Pond), the genus *Desulfomonile* (higher in abundance in Ephs Pond), and the genus *Dechloromonas* (higher in abundance in Ephs Pond).

There were 73 biomarkers for organic carbon source, fewer than for depth or sediment source (Figure 5C and Table S2). These biomarkers were mainly at lower taxonomic levels than those seen for depth and sediment source. The few phylum-level biomarkers for organic carbon source included Tenericutes (higher in columns created with kitchen food scraps and dominated by the genus *Acholeplasma*), Synergistetes (low in abundance but marking kitchen food scraps columns), and Actinobacteria, a biomarker for leaf litter columns.

**Figure 7:**
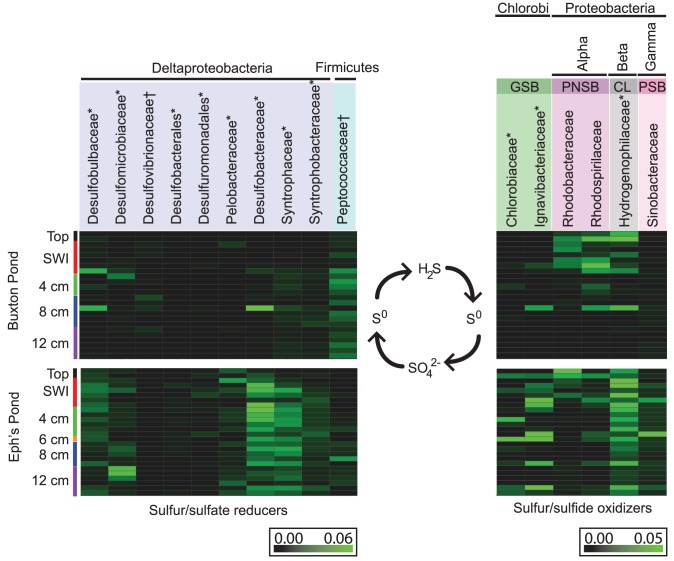
Sulfur cycling organisms identified in Buxton Pond and Eph's Pond Winogradsky column communities. Heat maps show the relative abundance of abundant sulfur and sulfate reducers (left) and sulfur or sulfide oxidizers (right). Samples are ordered by depth from top to bottom. *Eph's Pond column biomarkers, †Buxton Pond column biomarkers. GSB: green sulfur bacteria, PNSB: purple non-sulfur bacteria, CL: chemolithotroph, PSB: purple sulfur bacteria.

As an example of a nutrient cycle involving microbial processes, we investigated the abundance and distribution of sulfur-cycling organisms in the columns. Several different sulfur and sulfate reducing and oxidizing taxa were found throughout both Buxton Pond and Eph's Pond columns (Figure 7). In Buxton Pond columns, the Family *Peptococcaceae* of the phylum Firmicutes was the most abundant sulfur reducer, and was a biomarker. In Eph's Pond, several sulfur/sulfate reducing Deltaproteobacteria were identified as biomarkers. Sulfur and sulfide oxidizers in the Phyla Chlorobi and Proteobacteria were found in columns from both sediment sources, but were overall more abundant in Eph's pond. The chemolithotrophic *Hydrogenophilaceae* and phototrophic *Ignavibacteriaceae* were biomarkers for Eph's pond.

### Comparison to Natural Communities

**Figure 8:**
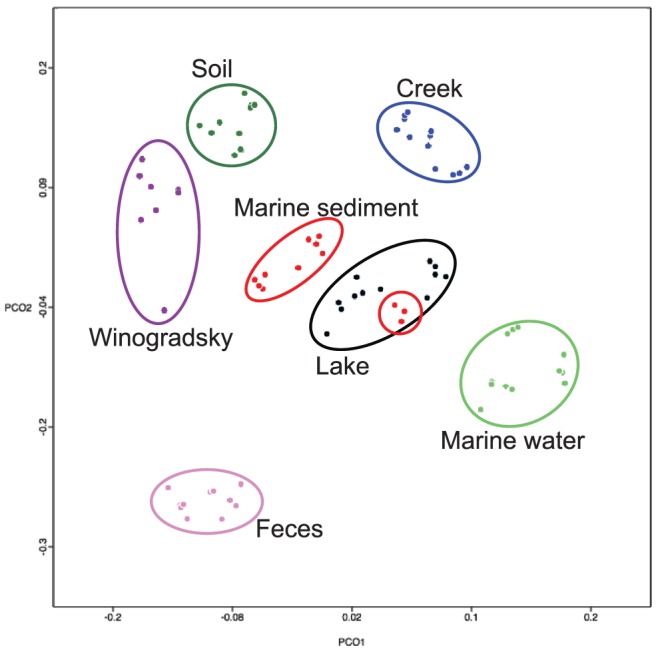
Comparison of Winogradsky column microbial communities to uncultured microbial communities. Samples from individual columns were pooled together to generate a single community for each column. MG-RAST was used to compare twelve samples from each of soil, marine sediment, creek, marine water, lake, and fecal biomes selected from previous metagenomics experiments (Caporaso et al.) targeting the V4 region of 16s rRNA (using single end sequencing of the 5′ end). These samples were compared to our data using the taxonomically-based Bray-Curtis index.

We wondered how Winogradsky column microbial populations compared to those seen in prior studies of uncultured microbial communities. We used MG-RAST to compare Winogradsky column communities to those found in several environments, including a freshwater lake, soil, and a creek [18]. Because our sequencing reads were longer and paired-ends were be assembled, we compared the Winogradsky column samples independently to both the 5′ and 3′ reads from the Caporaso *et al* study. We found that Winogradsky column microbial communities clustered separately from other biomes, but tended to be closer to soil samples than to any other samples in principal coordinate plots (Figure 8 and data not shown).

## Discussion

### Winogradsky column community structure

Winogradsky column microbial populations can be exceptionally diverse. As many as 30 phyla and 323 genera were present in an individual column, and among all columns 31 phyla and 414 genera were identified. Similarly high diversity in taxonomic groups has been observed in past studies of sediments, including over 40 phyla identified in a study of salt marsh sediments, and 18 phyla identified in a suboxic freshwater pond [29], [30]. The Shannon index also indicated an exceptionally diverse community, falling between 6 and 7 for SWI and deeper layers. This level of diversity is not unprecedented but is notable; a prior study of salt marsh sediment microbial communities indicated Shannon index values of over 7 [31]. Furthermore, because the rarefaction curves reach a plateau, our sequencing effort was sufficient to effectively capture the diversity of the samples even at low sampling depth.

Our results show that these Winogradsky column communities contain few highly abundant taxa and a large number of more rare microbes. Most genera identified in the Winogradsky columns were present at less than 1% relative abundance, while between 19 and 30 genera were found at greater than 1% relative abundance. The Berger-Parker index demonstrated that community dominance was similar among all subsurface samples, and rarefaction curves of OTU richness continued to rise with additional sequences per sample (Figure 3). This distribution of few abundant and numerous rare microbes is typical of soil and other microbial communities studied using high throughput techniques [32]–[36]. In some cases, rare microbes have been cultured or shown to be functionally important in the ecosystem [37], [38]. However, the concept that microbial communities contain a “rare biosphere” is controversial, as several studies have suggested that measurements of the rare biosphere, and therefore diversity, are inflated by sequencing errors [39]–[43]. Indeed, with increasing sampling depth and constant frequency of sequencing error, one would expect richness and singleton OTUs to continue to increase indefinitely due to error alone. The diversity of very low abundance OTUs therefore could be explained equally well by either rare microbes, sequencing artifacts, or a combination of both. We attempted to minimize the impact of sequencing errors on our data by clustering OTUs at 97% identity (rather than 100%), and quality filtering our sequences using both chimera detection and removal of extremely rare sequences. The remaining OTUs occurred in at least 2 samples and were represented by at least 50 sequences, which makes it less likely that these OTUs were the products of individual sequence errors or sequencing artifacts.

### Effect of environmental variables on Winogradsky microbial community

Our results suggest that the composition of the community in a Winogradsky column is shaped by a founder effect followed by diversification in stratified niches. When sediment is collected from the pond site, a founding population is captured and poured into the column. The high diversity of soil and sediment communities ensures that there are microbes that can thrive in the variety of niches that result in the column, including gradients of oxygen and hydrogen sulfide along the depth of the column. It is likely that the chemical and physical properties of the sediment contribute to the establishment of these niches as well. Colonization of the human microbiome is also subject to a similar founder effect followed by diversification. Delivery mode (vaginal vs. Cesarean section) influences the structure of the founding population that colonizes the infant [44], and over time site specific communities develop on the skin, gut, and other niches on or in the human body [45].

In our study, we found evidence of the founder effect on Winogradsky communities, as sediment source was an important driver of the composition of the resulting community. Beta diversity measures showed that regardless of depth or organic carbon source, columns made from the same sediment source were more similar to each other than columns made from different sediment sources (Figures 3 and 4). Sediment source biomarkers indicated that the same niches in Winogradsky columns may be filled by phylogenetically distant microbes according to a founder effect. Overall, there were more biomarkers for Eph's Pond than for Buxton Pond columns (Figure 5B), consistent with our alpha diversity results, which showed that Eph's Pond columns contained more diverse and rich microbial communities. Anaerobic members of the Proteobacteria, including sulfur cycle anaerobes within the Deltaproteobacteria, were higher in abundance in Eph's Pond columns at all layers than in the same layers of Buxton Pond columns. By contrast, the anaerobic phylum Firmicutes (including sulfate reducers within this phylum) was higher in abundance in Buxton Pond columns than Eph's Pond columns. These patterns suggest that both columns contained anaerobic and sulfur cycle niches dominated by different microbes according to a founder effect.

Once that founding population is added to the column, depth, and to a lesser extent, organic carbon source, allow the growth of specific bacteria in the different niches that are created. At the very top of the columns, the community, dominated by Cyanobacteria, was the least diverse and least rich (Figure 2). Samples collected by drilling into the SWI were the most variable but were no more diverse or rich than samples from greater depth. This indicates that, despite depth-based shifts in environmental conditions in Winogradsky columns, microbial diversity at all subsurface points remains high. Because we don't have information on the structure of the pond microbial community or the organic matter content of the sediments, we are unable to explain why supplementation caused only a minor shift in the population. The added leaf litter or vegetable scraps may have provided too little supplemental carbon to make a sufficient difference, or both sources may have provided similar supplements, changing the population from the founding pond sediments but not from eachother.

Using beta diversity analyses, we found separation of the phylogenetic and taxonomic composition of the communities by depth (upper layers: top surfaces and SWI, and lower layers: 4 cm, 8 cm, 12 cm). The separation of surface and SWI samples from deeper samples was more pronounced in weighted UNIFRAC, where relative abundance is taken into account. Weighted UNIFRAC has been suggested to be more appropriate in highlighting community differences based on shifts in abundance, such as those associated with differences in metabolite concentration [24]. We therefore propose that the differences seen between upper-level and lower-level samples reflect the major shifts in microbial community composition and structure that occur as conditions shift from oxic to anoxic. Similar patterns have seen in ponds receiving abundant organic matter and in flooded paddy soils, in which anoxic conditions rapidly develop close below the surface and anaerobic microbes produce methane and H_2_S [3], [29]. It will be interesting to determine if similarly high diversity as well as depth and sediment source effects are seen in Winogradsky columns prepared with materials from more distinct environments.

### Composition of Winogradsky column communities

Proteobacteria, Firmicutes and Bacteroidetes made up more than 75% of the community of each sample. Proteobacteria were highest in abundance at the tops of columns and decreased in abundance with increasing depth. Proteobacteria are frequently identified as an abundant member of sediment microbial populations, and Alphaproteobacteria and Betaproteobacteria have been linked to oxic zones of vertical oxygen gradients in previous studies [3], [29], [46]–[48]. Our work shows that this association of members of the Proteobacteria with oxic zones is duplicated in Winogradsky columns. Interestingly, in both Eph's and Buxton Pond columns, in lower-depth samples, the communities were either abundant in Proteobacteria or Firmicutes, but not both, suggesting that local conditions favor growth of one or the other.

Firmicutes and Bacteroidetes were low in abundance at the tops of columns and increased in abundance with depth. Firmicutes, mainly Clostridium Cluster one, has previously been associated with anoxic zones in vertical oxygen gradients [3], [46] and the genus *Clostridium* has long been described as an abundant member of the bottom layers of Winogradsky columns. The high abundance of the class Clostridia at lower layers supports past culture-based associations between Clostridia and anoxic zones of sediment.

Bacteroidetes is another anaerobic phylum and its increasing abundance with depth is likely a reflection of decreasing oxygen concentration with depth. Certain members of the Phylum Bacteroidetes are capable of degrading complex organic compounds like cellulose [49] and chitin and are likely key to the carbon cycle in the Winogradsky column. The simpler carbon compounds produced by these decomposers can be utilized by other fermenting organisms. Overall the distribution of aerobic and anaerobic groups at different depths reinforces the implications of beta diversity analyses and strongly suggests a major impact of oxygen concentration on microbial communities in Winogradsky columns.

The sulfur cycle is a key biogeochemical cycle that is driven primarily by microbial processes. The Winogradsky column has been used to enrich for sulfur cycle organisms, and used as a teaching tool for demonstrating the sulfur cycle. High-throughput sequencing of Winogradksy columns allows for detailed analysis of the ecology and spatial distribution of sulfur cycle microbes and provides an example of taxonomically distant microbes filling the same niches in microbial communities. Dissimilative reduction of sulfur compounds is carried out by sulfur reducing bacteria for energy conservation. Anaerobic respiration of sulfate (SO_4_^2−^) generates hydrogen sulfide (H_2_S), which has a distinctive odor and spontaneously forms ferrous sulfide with iron, visible as a black coloration of the soil. The sulfur reducing bacteria are not a monophyletic group, but rather are defined physiologically. Many sulfur reducers are in the class Deltaproteobacteria, but some Firmicutes and Archaea are also capable of sulfate reduction [50]. In these columns, the particular sulfur/sulfate reducers found differed by sediment source used. The sulfate reducing Firmicutes in the family *Peptococcaceae* were identified as a biomarker for Buxton Pond columns while other sulfur/sulfate reducers were rare or absent (Figure 7). In Eph's Pond columns, sulfur/sulfate reducing organisms were more diverse and abundant and were almost exclusively Deltaproteobacteria, most of which were biomarkers for Eph's Pond columns (Figure 7). Deltaproteobacteria are commonly found in sediment communities and have been identified as an abundant member of black layer communities in wetlands [29], [51], [52]. The family *Desulfobacteraceae* was the most abundant sulfate reducer and was distributed throughout Eph's Pond columns. In the family *Syntrophaceae* about one third of the sequences classifiable to a genus belonged to *Syntrophus*, which cannot reduce sulfate; however other sequences may belong to sulfate reducing members of this family. The *Syntrophobacteraceae* family is also comprised of a mix of sulfur reducers and non-sulfur reducers. The differences in sulfur/sulfate reducing communities between the two sediment sources demonstrate the functional overlap between highly divergent microbial groups.

Sulfur oxidizers complete the cycle by oxidizing H_2_S to elemental sulfur (S^0^) and SO_4_^2−^ through phototrophy or chemolithotrophy. The Purple Sulfur Bacteria (PSB) use H_2_S, and sometimes other reduced sulfur compounds, as an electron donor in photosynthesis. They have red, orange, blue and yellow pigments, resulting in the red-violet zone of the Winogradsky column, in the upper-middle portion of the column. The process generates elemental sulfur that is stored in intracellular globules and can be later oxidized to SO_4_^2−^. The PSB are all members of the class Gammaproteobacteria. Like the PSB, the Purple Non-sulfur Bacteria (PNSB) use H_2_S in photosynthesis, but generally have lower H_2_S concentration optima than the PSB. These are members of the Alphaproteobacteria and Betaproteobacteria. The Green Sulfur Bacteria (GSB) use H_2_S as an electron donor in photosynthesis, generating S^0^ and SO_4_^2−^. They are strict anaerobes, and are typically found in the “green zone” located in the lower-middle of the Winogradsky column. Neither the PSB nor the PNSB were found to be abundant in these columns (Figure 7). Although the GSB were more abundant, they were distributed unevenly in samples throughout the column rather than in the expected lower-middle zone. This is not surprising given that distinct green and red-violet zones were not apparent in these columns, although patches of color were present throughout at different intensities. Further, our sampling technique (drilling into the side of the column) may have excluded or destroyed surface-attached members of the microbial community.

Sulfur oxidation can also occur through chemolithotrophy, the use of a reduced sulfur compound as an electron donor in aerobic or anaerobic respiration. The chemolithotrophic sulfur oxidizer *Thiobacillus* was fairly abundant in the column, but especially in the upper-middle zones. They are shown in Figure 8 as *Hydrogenophilaceae*; the vast majority of the sequences identified in this family belong to the genus *Thiobacillus*. Another important chemolithotrophic sulfur oxidizer is the filamentous non-photosynthetic *Beggiatoa*, [53]. This species is strongly associated with Sergei Winogradsky, as its isolation and characterization by Winogradsky led to the concept of lithoautotrophy [54]. No *Beggiatoa* sequences were found in these columns.

We also identified a collection of microorganisms in the 4 cm and 8 cm layers involved in methane cycling. The phylum Euryarchaeota, one of the two Archaeal phyla detected in the columns, was a biomarker for samples at 8 cm. A prior study of microbial community composition in suboxic freshwater ponds identified Euryarchaeota as the only archaeal phylum in the sediment [29]. Euryarchaeota includes methanogenic microbes. Interestingly, the methanotrophic Methylococcales (a Gammaproteobacteria) was a biomarker for 4 cm samples, suggesting that methane produced in deeper layers is being used by methanotrophs above. The detection of Methylococcales in this layer is interesting, given that the black coloration of the sediment (indicative of metal sulfide precipitation) suggests an anoxic environment.

### Natural history of Eph's and Buxton Ponds

Both Buxton Pond and Eph's Ponds are located near Williams College, Willamstown, MA. Buxton Pond is entirely shaded and receives a substantial amount of leaf litter, while Eph's pond receives more direct sunlight. Buxton Pond is also closer to small roads. Both ponds have in the past experienced exposure to human waste. A sewer rupture in 1994 allowed raw sewage to flow into Eph's Pond for several days; no remediation was performed. Until the mid-1990s Buxton Pond was the receiving water body for one of the Buxton boarding prep school's cesspools. Several fecal microbiome microbes were biomarkers for Buxton Pond (*Bacteroides*, Enterobacteriales, and *Ruminococcus*), perhaps reflecting past fecal contamination. However, these are also cellulose degraders, and may be present in greater abundance than in Eph's Pond due to the differences in the amount of leaf litter that falls on the ponds. Columns from both ponds have microbes associated with contamination with aromatic organic compounds (*Dehalobacter*, *Desulfomonile, and Dechloromonas*), consistent with their history and location in a lightly developed municipality.

### Winogradsky columns as model communities

Microbial communities contribute critically to biogeochemical cycles, bioremediation, alternate fuel production, primary productivity, and numerous other processes critical in supporting ecosystems. They are also extraordinarily diverse and complex, containing numerous rare microbes, and are shaped by an enormous variety of factors including the presence or absence of other microbes, metabolic conditions, temperature, and pH. Studies of microbial communities in natural sediments are difficult to control for the impact of such variables. Here, we demonstrate the potential of Winogradsky columns for applying 16 s rRNA sequencing to the study of complex microbial communities. Winogradsky columns are easy to create, replicate, and manipulate, which allows for a degree of control not possible in field studies. We have demonstrated that Winogradsky column communities can be exceptionally diverse and display consistent patterns in microbial abundance based on depth, similar to natural ecosystems. Columns could also be manipulated to mimic the effects of changing temperatures, pollution, drought, or other effects relevant to current environmental challenges. Supplementing columns with different metabolites would offer a simple method for elucidating the effects of individual metabolites on stratified microbial communities. Time-course studies would offer insights in to the dynamics of communities over time as well as questions of succession and competition in sediment microbial communities. Winogradsky columns are self-contained and manipulatible ecosystems of diverse microbes and in combination with high-throughput sequencing could become a powerful tool for studies of microbial ecology.

### Winogradsky columns in undergraduate education

Winogradsky columns are commonly used in undergraduate microbiology courses, for example as tools to teach principles of nutrient cycling [55]. Metagenomics is an increasingly important part of microbiology, and together, the two present a unique opportunity to introduce students to ground-breaking technology, laboratory techniques such as extraction of DNA and PCR, and analysis of large, complex datasets. Several successful programs have already integrated education and genomics research [56]. Some of the work presented in this paper was performed within introductory microbiology courses at Vassar College and Williams College, and we envision that short or more extensive units on Winogradsky metagenomics could be an effective component of undergraduate microbiology courses, or could be the focus of an advanced undergraduate course on microbial ecology or metagenomics. We have developed teaching materials to guide teachers on using wet-lab and bioinformatic techniques for Winogradsky metagenomics in their courses [57], and emphasize that the data generated in these studies are a publicly available resource that can be used to supplement laboratory instruction.

## Supporting Information

Figure S1 **Relative abundance of phyla in Winogradsky columns.** A) Relative abundance in all samples, B–E) in samples at each depth, and F, G) in samples from the two mud sources. Vertical dashed lines demarcate phyla present at >1%, 1%–0.1%, and <0.1%.
(DOC)

Table S1 **Sample descriptions and identifying information.**
(XLS)

Table S2 **Winogradsky column biomarkers.**
(XLS)
