# Supplementary material for: 16S rRNA Gene Survey of Microbial Communities in Winogradsky Columns
Source: PLoS One. 2014 Aug 7;9(8):e104134. doi: 10.1371/journal.pone.0104134 (PMC4125166; doi:10.1371/journal.pone.0104134)
Supplement: Figure S1 — Relative abundance of phyla in Winogradsky columns. A) Relative abundance in all samples, B–E) in samples at each depth, and F, G) in samples from the two mud sources. Vertical dashed lines demarcate phyla present at >1%, 1%–0.1%, and <0.1%. (DOC) [file pone.0104134.s001.doc]

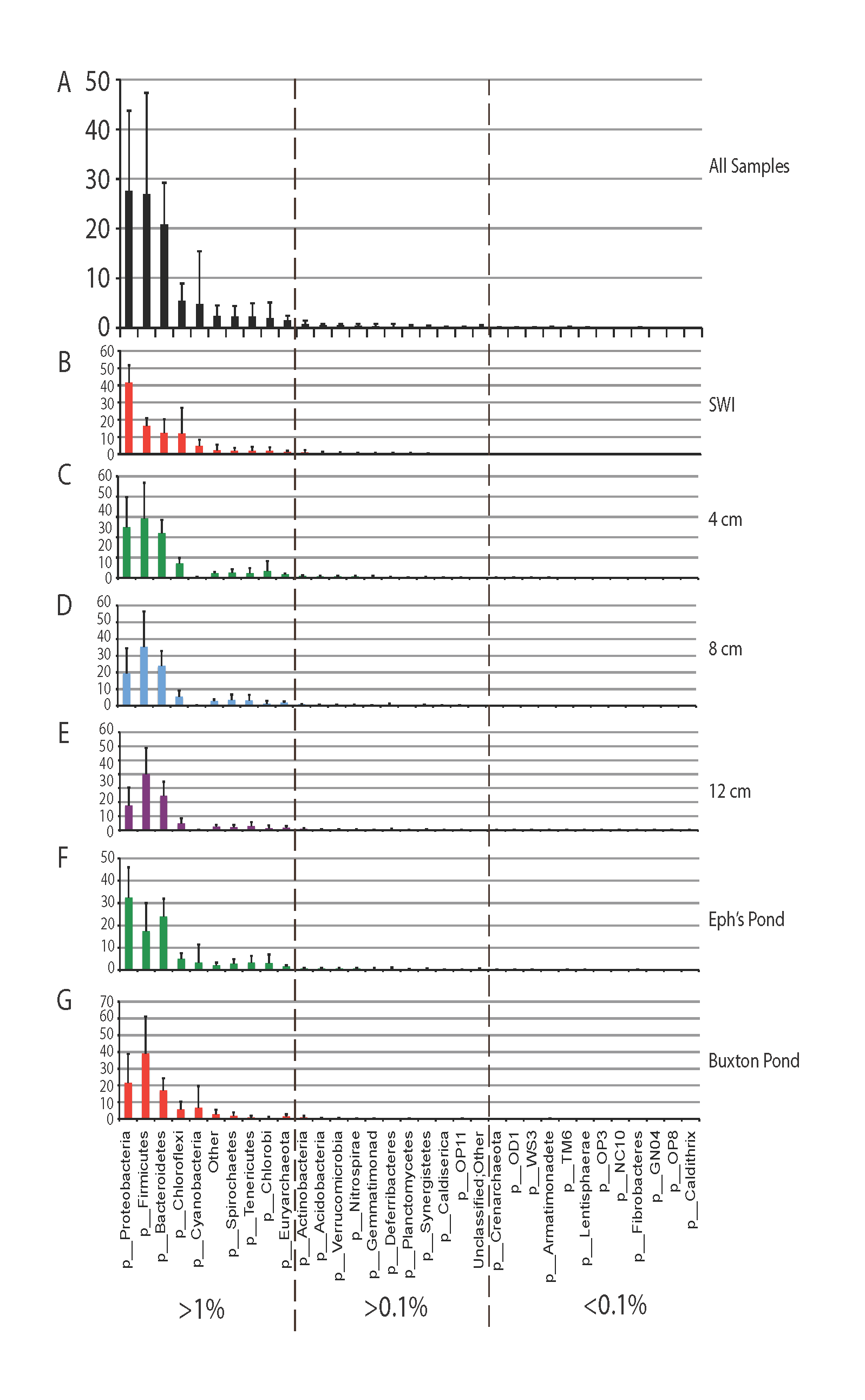


**Supplemental Figure 1: Relative abundance of phyla in Winogradsky columns.** A) Relative abundance in all samples, B-E) in samples at each depth, and F,G) in samples from the two mud sources. Vertical dashed lines demarcate phyla present at >1%, 1%-0.1%, and <0.1%.
